# The prognostic role of ultrasound and magnetic resonance imaging in obstructive sleep apnoea based on lateral oropharyngeal wall obstruction

**DOI:** 10.1007/s11325-022-02597-z

**Published:** 2022-03-30

**Authors:** Viktória Molnár, András Molnár, Zoltán Lakner, Dávid László Tárnoki, Ádám Domonkos Tárnoki, Zsófia Jokkel, László Kunos, László Tamás

**Affiliations:** 1 Department of Otolaryngology and Head and Neck Surgery, Semmelweis University, Szigony u. 36., 1083 Budapest, Hungary; 2 Faculty of Food Science, Hungarian University of Agriculture and Life Sciences, Gödöllő, Hungary; 3 Medical Imaging Centre, Semmelweis University, Budapest, Hungary; 4 Institute of Pulmonology, Törökbálint, Hungary

**Keywords:** Obstructive sleep apnoea, Ultrasound, Lateral pharyngeal wall, MRI, Drug-induced sleep endoscopy

## Abstract

**Purpose:**

This study examined the prognostic value of the lateral pharyngeal wall (LPW)-based obstruction and obstructive sleep apnoea (OSA) prediction using ultrasound (US) and MRI (magnetic resonance imaging).

**Methods:**

One hundred patients with and without OSA were enrolled, according to overnight polysomnography. The LPW thickness (LPWT) was measured using a Philips Ingenia 1.5 T MRI device, and US measurements were carried out at rest and during Müller’s manoeuvre (MM) with a Samsung RS85 device. The obstruction was localised under drug-induced sleep endoscopy.

**Results:**

Significantly greater LPWT using MRI was observed in the OSA group compared to the control group, while US results showed a significant difference only in the case of LPWT during MM on the left side. Obese patients presented significantly higher LPWT values. A significant correlation between BMI and LPWT was observed. Men presented significantly higher LPWT MRI values and left-sided LPWT using US compared to women. LPWT and AHI parameters were significantly correlated. The severity of LPW obstruction correlated with LPWT, while the LPW collapse significantly correlated with AHI. The severity of LPW collapse differed depending on the AHI values. Using US LPWT values and anthropometric parameters, a 93% effectiveness in OSA prognostication and 89% in LPWT-based obstruction were detected. MRI detected OSA in 90% and LPW-based obstruction in 84%. US successfully detected LPW-based collapse severity in 67%.

**Conclusion:**

US LPWT measurements were helpful in detecting OSA and LPWT-based obstruction. These examinations may be useful for surgical planning.

## Introduction

Obstructive sleep apnoea (OSA) is a leading public health problem due to its increasing prevalence [1]. OSA is characterised by total or partial collapse of the upper airways during sleep [2], which results in hypoxia, hypercapnia, and sleep fragmentation [3]. Characteristic symptoms are daytime sleepiness, fatigue, and concentration problems, though the disorder is undiagnosed in many cases. Based on a previous study, about one billion people of the 30–69 age group are suffering from the disorder worldwide [4]. In unattended cases, comorbidities, such as cardiovascular diseases [5, 6], stroke [7], and metabolic disorders [8] are present, indicating the importance of early diagnosis and therapy. The possible causes for OSA can be defined as an anatomical background, an ineffective function of the dilatator muscles, respiratory control instability (high loop gain), and low arousal threshold [9]. Overnight polysomnography (PSG) is recognized as the ‘gold standard’ for the diagnosis [8], while the anatomical localisation of the obstruction can be determined using drug-induced sleep endoscopy (DISE), which is also capable of detecting dynamic changes during sleep in three-dimensional space [10].

The lateral pharyngeal wall (LPW) consists of several muscles, which play a role in the velum and the tongue movements. Together with the lymphatic and adipose tissues, these muscles can be responsible for the obstruction [11]. The development of the soft tissues leads to the compression of the upper airways; therefore, the antero-posterior diameter takes an ellipse shape, which results in total obstruction of the LPW during sleep [12]. Müller’s manoeuvre (MM) is used to stimulate the upper airway obstruction in awake subjects. During this manoeuvre, the LPW lateral movements and the tongue antero-posterior motions of the conduct the change of the diameter of the upper airways. However, there is only a moderate correlation between this method and OSA severity [13]. Regarding medical imaging, computed tomography (CT), magnetic resonance imaging (MRI), and X-ray cephalometry are widely used. Ultrasound (US) imaging is not widespread in the diagnosis of OSA. There are only a few studies available of the use of US imaging in OSA. Although US examinations are subjective, these examinations may provide important information in cases with suspicion of OSA. For instance, Miller et al. have examined the motion of the LPW using US in a B/M mode to detect the effectiveness of swallowing therapies in healthy subjects [14]. Kim et al. have concluded that the US examination of the LPW in patients after stroke can be an alternative way to examine the pharyngeal phase of swallowing [15]. However, there is little knowledge regarding the prognostic value of the LPW assessment using radiologic imaging in OSA.

Therefore, the current study aimed to examine the LPW using US and MRI measurements. Based on our hypothesis, a combination of anthropometric measurements, demographic factors, and US imaging may help screen patients for possible OSA and LPW based obstruction.

## Materials and methods

### Subjects

In this prospective investigation, 100 adult patients (74 male and 26 females, mean age $\pm$ SD, 42.15 $\pm$ 11.7 years) were enrolled. The study was carried out at the Department of Otolaryngology and Head and Neck Surgery and Medical Imaging Centre of Semmelweis University. The examination of the patients consisted of a detailed case history, otorhinolaryngological examinations, overnight PSG, cervical US, and MRI examinations as well as DISE. Inclusion criteria were: snoring or possible OSA in the case history, patients over 18 years of age, and a written informed consent. Exclusion criteria included any previous otorhinolaryngological or oral surgeries, facial trauma, abnormalities of the soft tissues, any craniofacial malformations, neurological and psychiatric disorders, hypothyroidism or hyperthyroidism, alcohol or drug abuse, pregnancy, claustrophobia, and any metal implants in the body (e.g., pacemaker, etc.).

The study was approved by the Hungarian Research Ethics Authority (National Institute of Pharmacy and Nutrition, approval reference number: 2788/2019). All patients gave their written informed consent.

### Sleep test

Overnight PSG measurements were carried out at the Institute of Pulmonology, Törökbálint, under medical control, using a SOMNOscreen Plus PSG (SOMNOmedics GmbH Germany) device. According to the American Academy of Sleep Medicine recommendation, apnoea is defined as a reduction of the nasal airflow of 90% or more for at least 10 s. In the case of hypopnoea, there is a 30% or higher reduction of airflow, accompanied by ≥ 3 oxyhaemoglobin desaturation or arousal. The severity of OSA is based on the apnoea-hypopnea index (AHI), which is calculated according to the apnoea/hypopnea events per hour [16]. Due to the relatively low number of subjects, our patients were divided into OSA (*n* = 64) and non-OSA (*n* = 36, control) groups.

### Drug-induced sleep endoscopy

DISE was carried out by an experienced otorhinolaryngologist. Before the examination, 0.1% ephedrine for depletion and 2% tetracaine for local anaesthesia was used. Vein stripping was performed for intravenous anaesthesia, and the equipment for possible intubation in necessary cases was kept on standby. The patients were lying in a supine position, and 1.5 mg propofol per kilogram was used as narcosis. Oxygen saturation and blood pressure were measured during the procedure, and the breathing movements were also examined. An Olympus endoscope with a 3.5 mm diameter was inserted through the nose until the larynx to perform the examination. To interpret the results, the VOTE classification was used [17]. The possible localization of the obstruction can be at the V = velum, O = oropharynx, T = tongue base, and E = epiglottis. The structure of the obstruction can be antero-posterior, lateral, and concentric. 0 means no obstruction, 1 means partial obstruction, 2 indicates total obstruction, while X means that the obstruction cannot be visualized [17].

### Ultrasound

US measurements were performed at the Medical Imaging Centre of Semmelweis University by an experienced radiologist, who was blinded to the results of the other examinations of the patients. The US imaging was conducted using a Samsung RS85 US Device (Samsung Electronics Co., Ltd., Seoul, South Korea), with a CA1-7A convex (1–7 MHz) transducer in a gray-scale B mode. The patients were lying in a supine position during the examination with their head extended. The transducer was placed on the lateral side of the neck to the line joining the ear tragus and the infraorbital margin. Hence, the parapharyngeal space can be visualised in an oblique coronal plane. The ipsilateral internal carotid artery was identified using a Doppler mode, and next to the artery, the LPW could have be found as an echogenic line. The lumen of the pharynx cannot be clearly assed because of its gas shadowing. The LPWT is defined as a distance between the internal carotid artery and the echogenic interface, measured in the oblique coronal plane. The LPWT was measured three times both at rest and during MM, and the results were averaged.

### MRI

Unenhanced neck MRI examinations were performed at the Medical Imaging Centre of Semmelweis University, using a Philips Ingenia 1.5 T MR device (Philips Healthcare, The Netherlands), with a slice thickness of 4 mm. No intravenous contrast agent was applied. The subjects were asked to avoid motions and swallowing as well as to breathe through their nose.

The sequences were acquired as follows: sagittal localiser on the mid-line, coronal and axial T2 STIR, coronal and 3D axial T1 TSE, axial DWI, and sagittal T2 TSE. The participants were positioned supine with the head in a neutral anatomical position. Examinations were carried out with awake subjects. The participants were instructed not to swallow during the scanning, keep their mouths closed, and breathe through their noses.

**Fig. 1:**
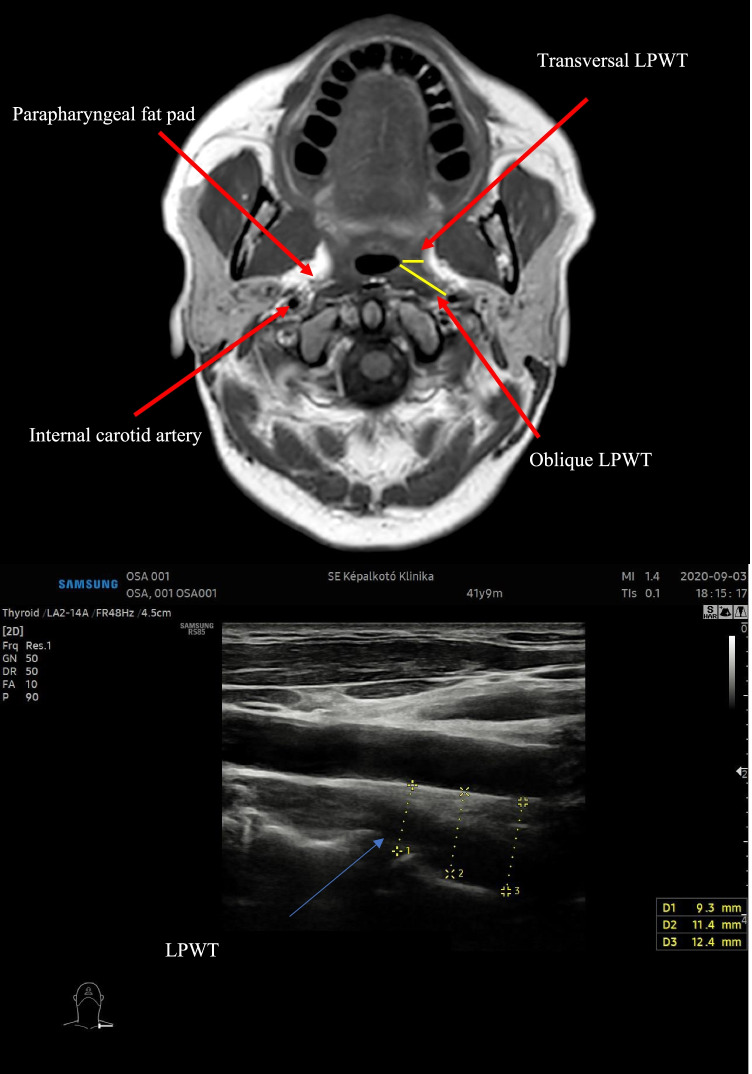
T1-weighed MR image (upper picture) of the LPW in the axial plane and US image (lower picture) in a gray-scale B mode. (Medical Imaging Centre of Semmelweis University). See the online version for coloured arrows

The transversal LPWT was defined as the distance between the medial border of the parapharyngeal fat pad and the lateral edge of the upper airways, measured in the images with the smallest airway cross-sectional area, using T1-weighed sequences. The oblique and transverse LPWT between the internal carotid artery and the lateral edge of the upper airways was measured using the same images. Measurements were performed on a Philips IntelliSpace portal (Philips Healthcare, The Netherlands) (Fig. 1).

### Statistical analysis

For data processing, different methods of automatic classification (e.g., logistic regression and discriminant analysis by StatCorp, 2015) and neural network (by Ciaburro and Venkateswaran, 2020) were applied. The multivariable analysis is a significantly developing area of statistics; therefore, there is no ‘gold standard’ for combining methods to achieve the highest level of specificity and sensitivity.

## Results

Table 1 shows the basic demographical data and the US and MRI parameters in the OSA and control groups.

**Table 1:** Basic demographical and US, MRI results of the patients. Parameters show the mean ± SD values. *** indicates a significant difference at *p* < 0.01 level, while ** indicates a significant difference at *p* < 0.05 level and * indicates a significant difference at *p* < 0.1. LPWT: lateral pharyngeal wall thickness. The bold emphasis indicates a statistically significant difference

Indicators	Control group (*n* = 36)	OSA patients (*n* = 64)	*p* value
Gender (male/female)	21/15	53/11	0.000***
BMI (kg/m^2^)	23.14 ± 3.87	30.99 ± 2.33	0.000***
Age (years)	38.13 ± 12.14	44.40 ± 10.9	0.000***
LPW US measurements			
LPWT on right side (mm)	6.38 ± 3.29	10.21 ± 4.55	0.142
LPWT on right side during MM (mm)	6.32 ± 3.30	9.98 ± 4.70	0.136
LPWT on left side (mm)	5.91 ± 3.13	8.78 ± 3.83	0.101
LPWT on left side during MM (mm)	5.95 ± 2.75	8.84 ± 3.18	0.068*
LPW MRI examination			
LPWT transverse thickness on right side (mm)	9.18 ± 2.65	10.67 ± 3.40	0.031**
LPWT oblique thickness on right side (mm)	19.81 ± 3.83	20.48 ± 3.84	0.462
LPWT transverse thickness on left side (mm)	9.45 ± 2.42	10.67 ± 3.49	0.067*
LPWT oblique thickness on left side (mm)	18.10 ± 4.12	20.11 ± 3.52	0.010**

Between the OSA and control groups, the gender, age, and BMI parameters of the patients were significantly different. Of US parameters only the left LPWT measured during MM was significantly different (*p* = 0.0679), although, there was a tendency of the additionally measured parameters to be higher in the OSA group. Using MRI, a statistically significant difference was observed in the case of the right transverse (*p* = 0.031), and in the case of the left transverse (*p* = 0.0671) and oblique LPWT, contrasted to the parameters of the control group.

The possible effects of gender, age, and BMI values on the US features of the LPW are summarised in Table 2.

**Table 2:** Correlation between US LPW parameters and anthropometric data of OSA patients. Parameters show the mean ± SD values. ** indicates a significant difference at *p* < 0.05 level and * indicates a significant difference at *p* < 0.1. The bold emphasis indicates a statistically significant difference

	Gender			Age (years)			BMI (kg/m^2^)			
	Male (*n* = 53)	Female (*n* = 11)	*p* value	≤ 40 years (*n* = 21)	> 40 years (*n* = 43)	*p* value	Normal (A) (*n* = 5)	Overweight (B) *n* = 24)	Obese (C) (*n* = 35)	*p* value
Right LPWT (mm)	9.76 ± 3.75	8.84 ± 6.9	0.528	10.2 ± 4.48	9.31 ± 4.37	0.453	5.2 ± 2.72	10.68 ± 3.99	9.5 ± 4.52	0.418
Right LPWT during MM (mm)	9.46 ± 3.92	8.19 ± 5.29	0.359	10.09 ± 4.52	8.83 ± 3.97	0.260	5.96 ± 2.95	10.43 ± 4.03	8.9 ± 4.17	0.837
Left LPWT (mm)	9.24 ± 3.48	6.81 ± 3.49	0.039**	9.76 ± 3.73	8.36 ± 3.45	0.142	5.29 ± 3.46	8.85 ± 3.3	9.3 ± 3.59	0.057 (A-C)*
Left LPWT during MM (mm)	8.83 ± 3.10	6.8 ± 3.19	0.054*	9.08 ± 3.65	8.19 ± 2.94	0.298	5.81 ± 3.58	9.23 ± 2.71	8.35 ± 3.3	0.592

In male patients, significantly higher values of the left LPWT were observed at rest (*p* = 0.03) as well as during MM (*p* = 0.05). Age had no significant effect on the parameters. According to BMI parameters, the left LPWT significantly differed (*p* = 0.05) between normal weighted and obese patients.

The possible effects of the gender, age, and BMI values on the MRI features of the LPW are summarised in Table 3.

**Table 3:** Correlation between MR LPW parameters and anthropometric data of OSA patients. Parameters show the mean ± SD values. *** indicates a significant difference at *p* < 0.01 level, while ** indicates a significant difference at *p* < 0.05 level. The bold emphasis indicates a statistically significant difference

	Gender			Age (years)			BMI (kg/m^2^)			
	Male (n = 53)	Female (n = 11)	*p* value	≤ 40 years (*n* = 21)	> 40 years (*n* = 43)	*p* value	Normal weight (*n* = 5)	Overweight (*n* = 24)	Obese (*n* = 35)	*p* value
Right LPWT (transverse) (mm)	11.13 ± 3.31	8.28 ± 2.91	0.011**	13.11 ± 3.42	9.43 ± 2.69	0.000***	12.54 ± 3.13	10.42 ± 3.09	10.51 ± 3.63	> 0.999
Right LPWT (oblique) (mm)	21.14 ± 3.66	16.82 ± 2.44	0.000***	22.49 ± 3.21	19.44 ± 3.77	0.002***	20.94 ± 0.55	19.13 ± 3.28	21.23 ± 3.84	> 0.999
Left LPWT (transverse) (mm)	11.21 ± 3.47	8.03 ± 2.36	0.000***	12.65 ± 3.64	9.65 ± 3.02	0.002***	10.81 ± 2.47	10.95 ± 3.42	10.44 ± 3.72	> 0.999
Left LPWT (oblique) (mm)	20.71 ± 3.43	17.23 ± 2.40	0.002***	21.73 ± 3.09	19.37 ± 3.49	0.011**	19.40 ± 4.34	19.11 ± 3.64	20.92 ± 3.21	> 0.999

In the case of male patients, significantly different values of transverse and oblique LPWT were measured on both sides. Under 40 years, significantly higher transverse and oblique LPWT parameters were registered. BMI had no significant effect on the MRI features of the LPW.

### Correlation between LPWT and AHI, BMI values

**Fig. 2:**
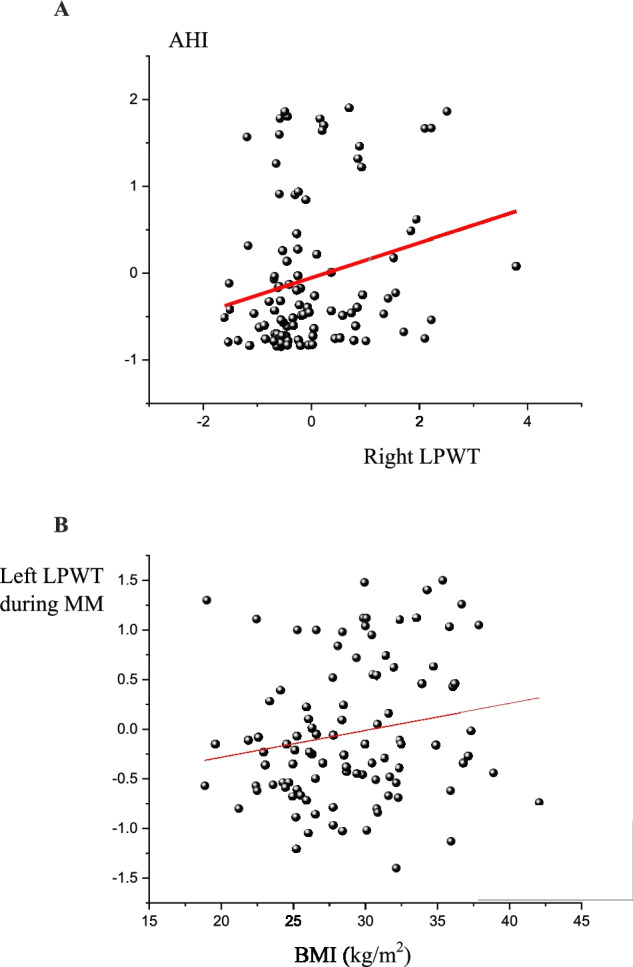
**A** Correspondence between LPW and AHI. **B** Correlation between LPWT and BMI. The figures show the transformed values of LPW and AHI

US LPWT correlations with AHI and BMI are shown in Fig. 2A and B.

As shown in Fig. 2, a slight but significant correlation was detected in the case of LPWT and AHI (*r* = 0.23, *p* < 0.05). LPWT and BMI were also correlated slightly, although, significantly (*r* = 0.18, *p* = 0.074).

### Correlation between LPW-based collapse and AHI, and LPWT

**Fig. 3:**
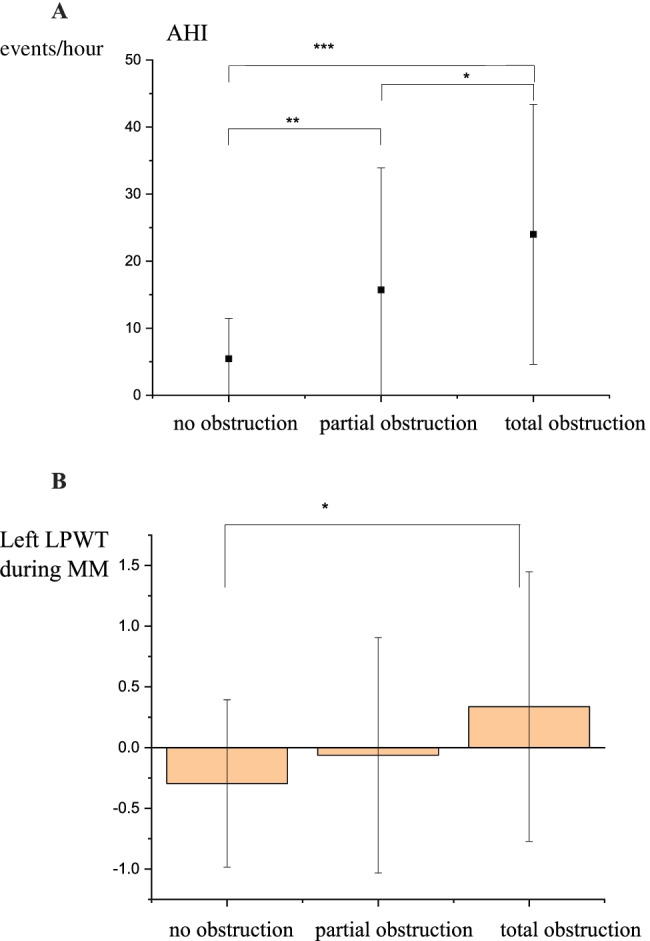
**A** Correlation between LPW based collapse and AHI. **B** Between LPW-based collapse and LPWT. The figures show the normalised (transformed) values of LPWT. *** indicates a significant difference at *p* < 0.01 level, while ** indicates a significant difference at *p* < 0.05 level and * indicates a significant difference at *p* < 0.1

The correspondence between LPW based collapse and AHI and LPWT are shown in Fig. 3A and B.

As Fig. 3 reveals, a significant correlation was observed between AHI and collapse severity, indicating the highest AHI values in the case of total obstruction. Depending on the AHI values, the LPW collapse severity significantly differed. Between LPWT and LPW-based obstruction, significant, non-linear correspondences were observed. The highest LPWT values were detected in the case of a total obstruction in this case as well.

Table 4 presents the outcome of the quadratic discriminant analysis of the classification results (sensitivity and specificity).

**Table 4:** Classification of OSA and LPW-based obstruction, according to gender, age, BMI, US and MRI parameters

	US measurements		MRI measurements	
	Estimated parameter		Estimated parameter	
	Obstruction	OSA	Obstruction	OSA
True positive	29	60	30	60
True negative	60	33	54	30
False positive	7	2	13	6
False negative	4	5	3	4
Indicators of binary classification (confusion matrix)				
Sensitivity	0.878	0.923	0.750	0.937
Specificity	0.895	0.942	0.814	0.833

As shown in Table 4, the most important indicators of the different methods were similar to each other, although, in some cases, the results of US can be used more effectively for the discrimination of OSA and LPW-based obstruction than MRI parameters.

A combination of US measurements of the LPW and anthropometric parameters can predict the presence of OSA and the LPW-based obstruction in 93% and 89%, respectively. In the case of MRI, these parameters were defined as 90% and 84%. The sensitivity of US and MRI for screening of OSA was similar (93%); however, a better specificity for US measurements (94% vs 83%) was observed. In the case of LPW-based obstruction, 87% sensitivity for US and 75% for MRI while 89% specificity for US and 81% for MRI were defined.

The estimation of obstruction severity based on US LPWT values using quadratic discriminant analysis is shown in Table 5A and B.

**Table 5:** **A** and **B** Determination of obstruction severity using US LPWT and gender, BMI, and neck and hip circumference parameters. The analysis was performed using quadratic discriminant analysis

A			
Real obstruction	Estimated obstruction		
	No obstruction	Partial obstruction	Total obstruction
No obstruction	30 (88%)	3 (9%)	1 (3%)
Partial obstruction	7 (27%)	18 (69%)	1 (4%)
Total obstruction	13 (32%)	6 (15%)	21 (53%)
B			
Real obstruction	Estimated obstruction		
	0/partial obstruction		Total obstruction
No/partial obstruction	60 (60%)		1 (1%)
Total obstruction	1 (1%)		38 (38%)

As shown in Table 5A, using US LPWT values and basic anthropometric parameters (i.e., gender, BMI, neck and hip circumferences), the categorization of obstruction severity was successful in 67%. The best matches were observed in those cases when no obstruction was detected (88%). In the case of partial obstruction, the results correlated in 69%, while the worse outcome was detected in the total obstruction group (53%). Table 5B shows the determination of obstruction severity using US LPWT and gender, BMI, and neck and hip circumferences. Quadratic discriminant analysis was used for data processing, and obstruction severity was grouped into two categories: no or partial obstruction and total obstruction.

As shown in Table 5B, when obstruction severity was divided into two categories, LPW-based obstruction was successfully prognosticated in 98%, using a neural net r-package, including a five neuronal network system.

## Discussion

The upper airway is a complex anatomical unit which plays a role in essential physiological functions, such as swallowing, speaking, and breathing. Medical imaging of this unit contributes to the examination of the pathophysiology in a more detailed manner; thus, the pathogenesis of OSA can also be better understood. US measurements of the head and neck region are frequently used for indications other than OSA screening in everyday clinical practice, since these examinations are fast, relatively unexpensive, also contain much information regarding the different anatomical regions. Although OSA is presented with typical daytime and overnight symptoms, the diagnosis and adequate therapy is lacking in many cases. Hence, quality of life impairment and comorbidities often occur. In the background of undiagnosed and treated OSA cases, infrastructure-related problems as a possible explanation can be identified, but the patients’ education is often lacking as well. Our study aimed to find a possibility to solve the problem mentioned above. Therefore, this study analysed the possible role of LPW US measurements to screen undiagnosed OSA cases, as a supplement of the generally used US examinations, along with the detection of LPW-based obstruction. The applied algorithm used the basic anthropometric parameters and US measurements of the LPW. In the current study, the LPW of OSA and control patients was examined, using US and MRI. To the best of our knowledge, it is the first investigation which analysed the prognostic value of US and MRI in patients with OSA and LPW-based obstruction.

The plane of the upper airways is different in normal subjects and in patients with OSA: in normal cases, the horizontal dimensions are dominant, while in the case of OSA, due to the lateral obstruction, the antero-posterior dimension is more expressed [18, 19]. Changes in the configuration of the upper airways during sleep lead to the occurrence of obstructions, which can be visualised using DISE. The categorisation of the obstruction is based on several classification schemes, although none of them differentiates the LPW-based obstruction [20]. In the current study, the VOTE classification was used, in which case the LPW is integrated into the oropharynx-based obstruction category.

Lan et al. have observed a strong relationship between the LPW-based obstruction and OSA severity [21]. According to the dynamic MRI results of Liu et al., a strong correlation was detected between LPW collapse and OSA severity [22]. Based on previous studies, the increase of the LPWT results in a rising tendency of apnoea events [23]. Based on our analyses, AHI and LPWT-based obstruction severity were significantly correlated, indicating the importance of LPW obstruction in OSA severity.

The assessment of the LPW can be carried out using CT, MRI, or US examinations. Using MRI in our study population, significantly higher transverse and oblique LPWT values were registered on both sides in the OSA group, which is similar to the results of Schwab et al., who have also observed higher LPWT by MRI measurements [11]. According to US imaging, only the left LPWT was significantly different in the OSA group, in contrast to the parameters of the control subjects. Hussein et al. have found significantly higher LPWT in case of moderate and severe OSA, using US examinations [24]. Bilici et al. have concluded that using submental US measurements, higher LPWT values of severe OSA patients can be registered than in the mild and moderate groups [25]. Liu et al. have observed a positive correlation between the LPWT and OSA severity [12]. Our results indicated a slight but significant correlation between LPWT and AHI values. The complex relationship between LPW-based collapse and LPWT was confirmed by the significant non-linear correlation between them. Therefore, it can be stated that besides anatomical factors, other parameters are also significant in the case of LPWT-based obstruction.

The different US and MRI features of the LPW reported in our study can be explained by the different planes and anatomical localizations of the examinations. Liu et al. have registered a good correlation between the LPWT measured by US and the transverse LPWT measured by MRI [12]; however, such a correlation was not found based on our results.

In male patients, a significantly higher thickness of the left LPW using US and higher transverse and oblique LPW values were observed on both sides using MRI. The higher LPWT of males can result in a higher occurrence rate of collapses; therefore, it can be responsible for the predominance of male patients in OSA. The thickness of the LPW of obese patients was significantly higher, contrasted to the parameters of the normal group, which can be explained by the adipose tissue accumulated in the muscles. A slight but significant correlation was observed in the case of LPWT and BMI. The accumulation of adipose tissue can result in a higher LPWT and increased risk for LPW-based collapses. Previous studies, for instance by Lan et al., have observed a significant positive correlation between BMI and LPW-based obstruction [21].

It is a well-known fact that obesity is one of the most important risk factors for OSA; however, its exact role in the pathophysiology of the obstruction has not been identified yet [26]. Based on previous studies, the adipose tissue in the parapharyngeal spaces increases with obesity, leading to upper-airway obstruction [27]. Other investigations highlight the role of the thickness of the LPW in the pathogenesis of OSA [11].

Based on our results, US examinations combined with anthropometric parameters (e.g., gender, age and BMI), the presence of OSA and LPW-based obstruction can be predicted in 93% and 89%, respectively. The sensitivity and specificity of US for OSA were defined as 92% and 94%, respectively, while these parameters for LPW based obstruction were calculated as 87% and 89%.

According to the statistical analysis, a combination of anthropometric measurements and MRI, OSA, and LPW-based obstruction can be predicted in 90% and 84%, respectively. The sensitivity of MRI in screening for OSA was defined as 93%, while 83% specificity was detected. These parameters in the case of LPW-based obstruction were 75% and 81%.

Our algorithm, including US LPWT and anthropometric parameters, was able to detect LPW based obstruction in 67%. The complexity of the background of LPW-based obstruction was verified by the fact that the prognostication by the algorithm was less accurate in the case of a total obstruction (i.e., 53%). This can be explained (besides the biases) by the non-anatomical risk factors for OSA (e.g., impaired dilatator muscle activity). Although in those cases when no obstruction was detected, the algorithm was accurate in 88%. When obstruction severity was categorised into two groups, the accuracy of the prognostication was defined as 98%. The LPW plays a vital role in the development of obstructions; therefore, it is essential in the severity of OSA due to LPW-based obstruction. The examinations of LPWT are significant for diagnosing OSA as well as therapy planning.

For instance, the determination of high pharyngeal collapsibility may be determined as a contraindication for surgical treatment.

LPW US examinations can be significant in diagnosing OSA and measuring LPW collapsibility. Our further study aims to examine more patients to analyse LPWT in a more detailed manner and generate a more accurate prognostication algorithm.

Our study has some limitations. First, due to the relatively low number of patients, we were not able to create more groups according to the severity of OSA. US measurements were carried out with the head of the patients in an extended position; therefore, the LPW can be easier visualised, and the acoustic shadowing of the mandible can be reduced. This position during the examination is advantageous, although it does not represent the anatomical situation during sleep. US and MRI examinations were carried out in awake subjects, although, the airway diameters could be analysed more precisely during sleep. During MM, pharyngeal manometry was not used; therefore, the measurements were not carried out uniformly. Before the DISE examinations, a depletion of the nasal mucosa was used, which can be a limitation as it can influence the collapse of the pharyngeal wall under sedation.

## Conclusion

Our results highlight that the US measurements of the LPW, as a supplement of the US measurements of the head and neck region generally used in everyday practice, can be used with high precision for OSA screening and LPW based obstruction grading, in previously undiagnosed cases of OSA. The results of the present investigation suggest that US evaluation of the LPW may be advantageous for patients with possible OSA, as it provides an inexpensive, fast, and low stress examination in everyday clinical practice.
